# A case report of lameness in two dairy goat herds; a suspected combination of nutritional factors concurrent with treponeme infection

**DOI:** 10.1186/s13104-015-1734-3

**Published:** 2015-12-16

**Authors:** Margit Groenevelt, Katharine Anzuino, Sue Smith, Michael R. F. Lee, Rosemary Grogono-Thomas

**Affiliations:** School of Veterinary Science, University of Bristol, Langford, Somerset, BS40 5DU UK; Willow Walk, Honiton, EX14 2FX UK; Castle Cary, BA7 7EG UK; Rothamsted Research, North Wyke, Okehampton, Devon EX20 2SB UK

**Keywords:** Dairy goats, Lameness, Treponemes, Nutrition

## Abstract

**Background:**

Two dairy goat farms with high level of lameness in lactating animals were presented for further investigation. Farm 1 and Farm 2 presented with 37 and 67 % morbidity, respectively. Both farms had an all year round indoor system, feeding ad libitum concentrate with forage available at all times.

**Case presentation:**

The lameness was found to be based in the foot. Previous treatments consisting of biweekly footbathing with zinc sulphate, spraying lesions with oxytetracycline spray and packing lesions with copper crystals on a single occasion and single injections with long acting oxytetracycline had not been successful. Mild cases had signs of haemorrhaging in the white line or on the sole of the foot. Moderate cases showed under running of the wall horn or small areas of exposed sole corium. Severe cases would consist of horn or wall separation with the corium exposed and infected. In extreme cases only the wall horn of the claw remained, with a large area of necrotic tissue in the centre and no healthy corium visible. Only one animal was seen to have interdigital lesions. Polymerase chain reaction (PCR) and culture of swabs taken from exposed corium and the interdigital space were negative for *Dichelobacter nodosus* but PCR for treponemes were positive in both the adults and the youngstock tested. Due to the high level of concentrate in the diet of these goats, nutrition was thought to contribute to the problem. Transcutaneous rumen fluid samples were taken and pH was measured on both farms, with 35 % of the samples below pH value 5.5.

**Conclusion:**

No definite diagnosis could be made. However, the results suggest both treponemes and nutrition play a role in the aetiology of the lameness. The initial sole or wall horn lesions were thought to be secondarily infected by treponemes. Further investigation is needed to definitively diagnose the cause and contributing factors for this lameness.

## Background

Lameness in sheep and dairy cattle has been extensively researched. It is clearly demonstrated that lameness in dairy cows is a painful condition [1] and has an influence on fertility [2], productivity [3] and longevity [4]. In sheep, lameness has been associated with weight loss [5], decreased fertility and lamb growth rates [6]. It is estimated that the average lameness level in the dairy cattle herd in the UK is 36 % [7] and for sheep this lies between 8 and 10 % [8, 9]. In contrast, very little research has been undertaken into lameness in dairy goats. Previous observations on dairy goat farms in the UK estimated the prevalence of lameness to be between 9.1 [10] and 19.2 % [11]. In sheep, 90 % of lameness cases are caused by footrot (*Dichelobacter nodosus*) [9]. *D. nodosus* has also been confirmed as a cause of lameness in goats [12, 13]. However, Hill et al. [10] found only one of four dairy goat farms investigated to be affected by footrot, with 14.2 % of animals showing lesions. There is little published evidence for high levels of footrot in UK dairy goat herds. However, in the authors’ experience, farmers often presume a high prevalence of lameness in their goats must be due to footrot infection. It is reported that up to 100 % of dairy goat farms may have some goats with overgrown feet and up to 91.7 % of farms have goats with severely overgrown feet (>2.5 cm) [11]. Severely overgrown or deformed feet are associated with higher mobility scores and claw temperatures in dairy goats and could therefore contribute to lameness levels on farm [14]. Other lesions found on UK dairy goat farms include horn separation, white line lesions, abscesses of the sole, foreign bodies, and granulomatous lesions [10]. In other countries, major lameness causes in goats include footrot [15], white line lesions, foreign bodies [16] and, in addition, foot abscesses and sole ulcerations [17].

In dairy cattle a link between ruminal acidosis due to a high non-structural-carbohydrate (concentrate) diet and claw horn lesions is often suggested. Although in cattle this relationship is well documented, there is still no conclusive evidence as to the causative pathogenesis [18]. In goats, there is no literature available that establishes a link between nutrition and lameness. There is some work published on different feeding regimes for intensive dairy herds [16, 19, 20] but none of these studies have been carried out for longer than 8 months during lactation or on large groups of animals. The impact of these intensive, high concentrate feeding regimes on animal health, including lameness, is currently unknown.

In recent years, new foot diseases associated with treponeme infection have been described. Treponemes have been suggested as the causative agent in contagious ovine digital dermatitis (CODD), causing severe lameness in sheep. Multiple strains/serotypes of treponemes have been identified in association with CODD [21]. These strains have some genetic similarity with those associated with bovine digital dermatitis (BDD), despite different clinical lesions and pathology [22]. More recently, non-healing white line lesions (NhWL) and sole ulcers (NhSU) in cattle have been reported to be associated with treponemes [22]. To date there has been one publication that reports the isolation of treponemes from goats’ feet from lesions that look similar to CODD [23]. This paper reports on lameness observations in two large dairy goat herds in the UK, possibly associated with nutrition and treponemes isolated from the lesions.

## Case presentation

### Farm visits

The investigation took place on two dairy goat farms in the South West of England. Both had been dealing with high levels of lameness for over four years. Intervention measures had focussed on treatment and prevention of footrot and had not resulted in a reduction of lameness. Their attending veterinary surgeons had referred these cases for further investigation. Each farm was visited at least three times throughout the investigation. A full history was taken from the farmers and a thorough inspection was carried out of the housing, milking parlour, bedding and feeding regime (see Table 1).

**Table 1:** Overview of farm details

	Farm 1	Farm 2
Number of lactating does	313	540
Breed	British Saanen, British Toggenburg	British Saanen, British Toggenburg, British Alpine
Housing	Straw bedding	Straw bedding
Feeding	Ad libitum concentrates Ad libitum grass hay or haylage	Ad libitum concentrates Ad libitum grass silage
Foot trimming	Every 6–8 weeks	Every 3 months
Foot bathing	Not carried out	Every 2–3 weeks (zinc sulphate)
Production level	1000 l/doe/year	1050 l/doe/year
Kidding regime	1 kidding/doe/year	1 kidding/doe/year
Parlour	Flat entry and exit	Sloped entry and exit

### Lameness scoring

Lameness assessment and scoring was carried out according to Anzuino et al. [11] as in Table 2 on all lactating animals during morning milking on both farms. Lameness was define as a score ≥1. The goats were scored after exiting the parlour on a flat, concrete passage way. Care was taken that the scorer did not obstruct the goats so normal behaviour could be seen. Youngstock (between 2 and 12 months of age) were not scored but were observed for lameness in their pens.

**Table 2:** Lameness score definitions [11]

Score	Definition
0	Goat places full weight on all four limbs, moves forward freely with an even gait
1	Goat has a definite limp on one or more legs, but bearing weight and moves forward freely
2	Goat has some difficulty moving forward, severe limp, bearing little weight on one or more legs, may be a degree of goose-stepping
3	Goat has some difficulty moving forward, non-weight bearing on one or more legs, or may ‘goose-step’ high or walk on the knees

**Fig. 1:**
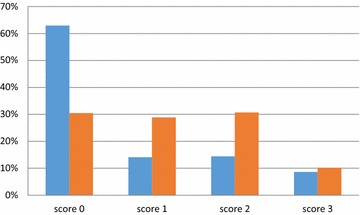
Lameness score results on Farm 1 (*red bars*) and Farm 2 (*blue bars*)

The lameness prevalences in the lactating animals were 37.1 % for Farm 1 and 69.6 % for Farm 2 (Fig. 1). No lameness was detected in the youngstock. Youngstock was kept in different housing to the adults on both farms although in close proximity. No biosecurity measures were in place between youngstock and the lactating herds.

### Clinical examination

The goats were clinically examined on two occasions on each farm, firstly during treatment sessions and later during routine foot trimming session. During each session, 15–20 goats were closely examined by the researchers. These animals were selected based on lameness score, lesion type or absence of lameness to ensure a full overview of the condition of the feet was acquired. All four feet of each animal were examined, regardless of which leg the animal had been lame on. Care was taken not to interfere with the normal trimming and treatment routines on either farm in order to assess the normal protocols.

**Fig. 2:**
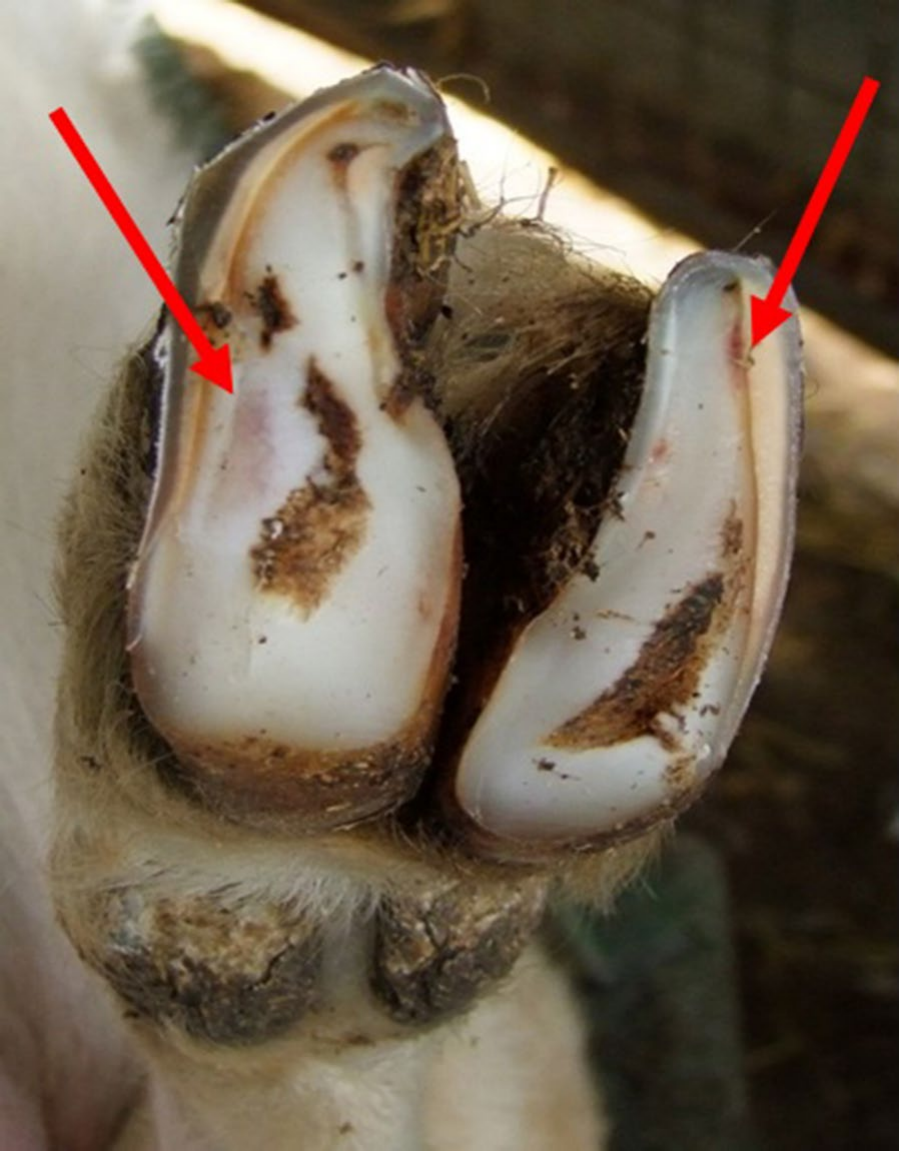
Mild lesion showing haemorrhage in the white line or the sole area of the foot (*see arrows*)

**Fig. 3:**
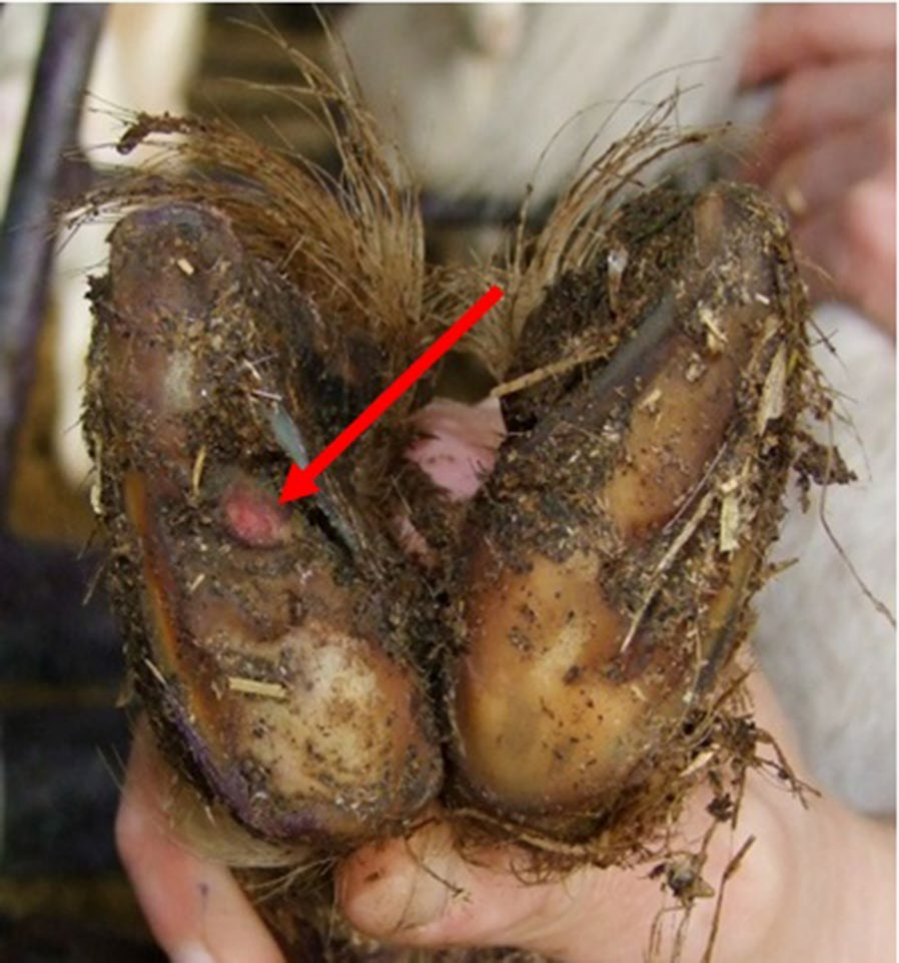
Moderate lesion showing a small area of corium exposed in the sole horn (*see arrow*)

**Fig. 4:**
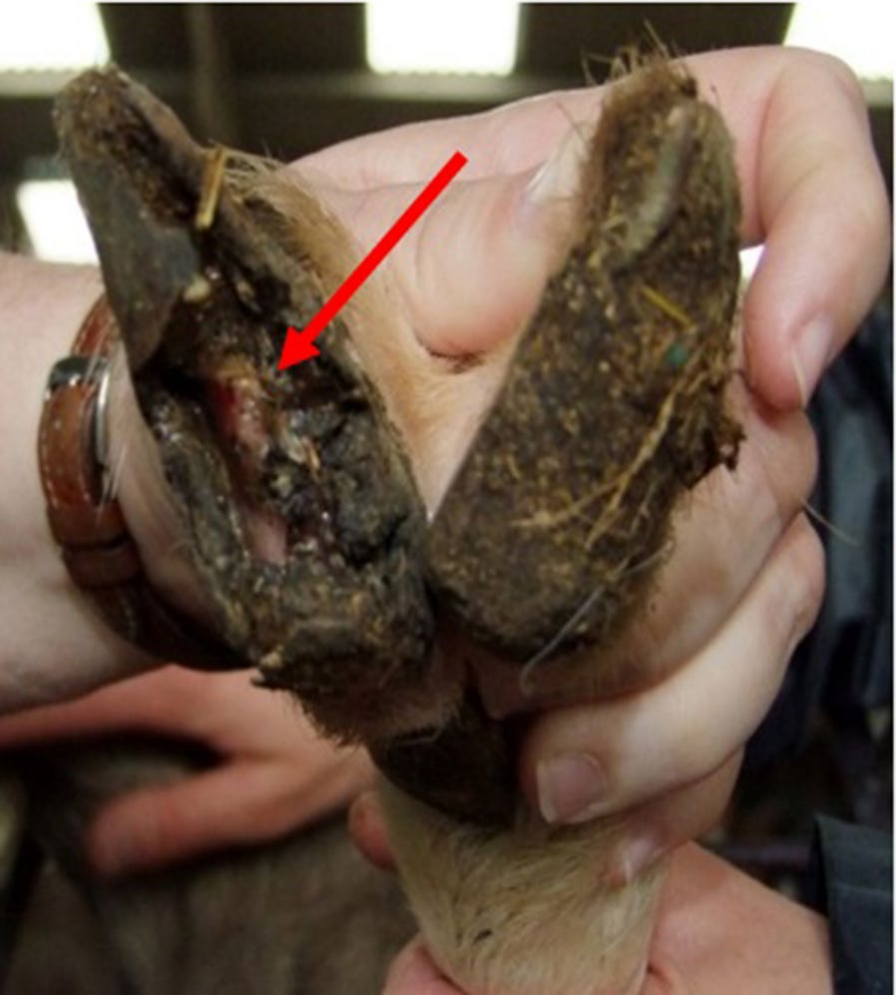
Severe lesion showing a large area of sole exposed with the underlying corium being infected (*see arrow*)

**Fig. 5:**
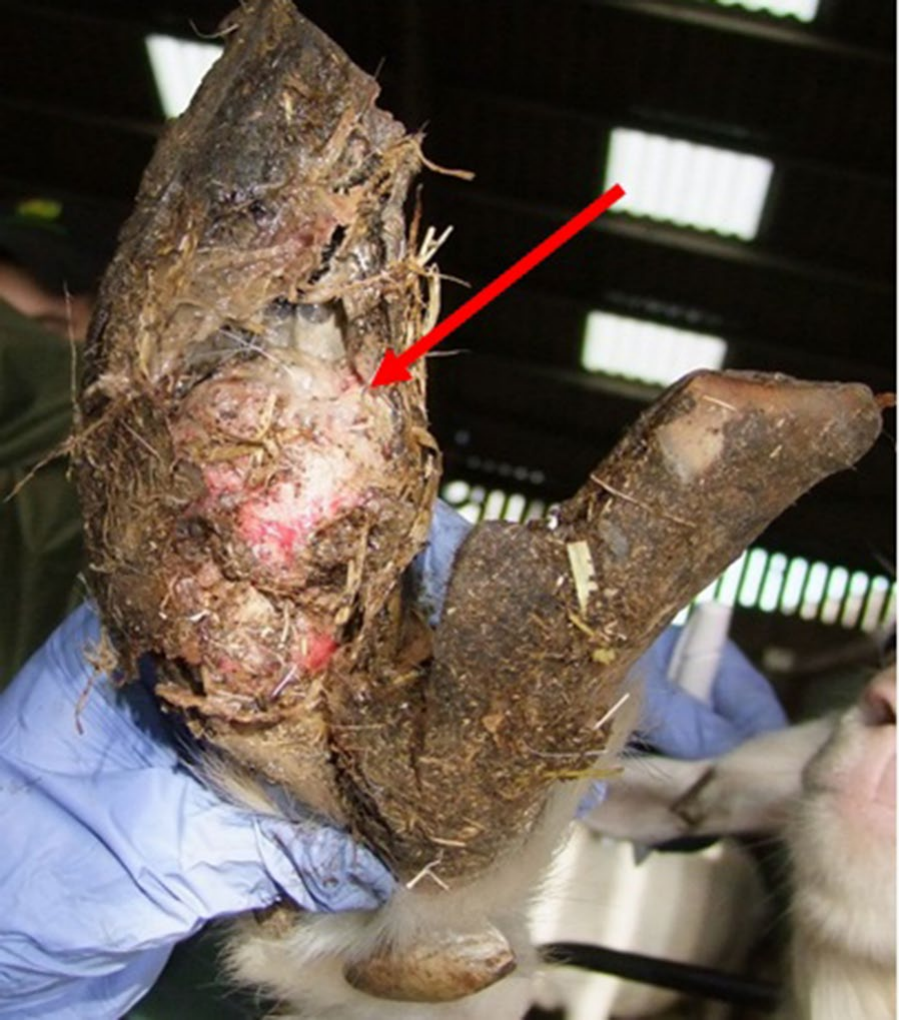
Extreme lesion showing only the wall horn of the foot remaining, leaving a large area of necrotic tissue with no healthy corium visible (*see arrow*)

During these sessions a variety of foot lesions were observed. The different lesions were classed as mild to extreme (Table 3; Figs. 2, 3, 4, 5). Lesions were most often seen in one leg and one claw but could affect both claws on the same leg or multiple legs. Only on one occasion were lesions in the interdigital space observed that resembled scald, the skin being moist, red and with a foul smell.

**Table 3:** Description of classification of lesion severity

Severity	Description
Mild	Haemorrhage in the white line or the sole area of the foot (Fig. 2)
Moderate	Under running of the horn of wall or sole, sometimes with small areas of corium exposed (Fig. 3)
Severe	Large areas of wall or sole exposed with the underlying corium being infected (Fig. 4)
Extreme	Only the wall horn of the foot remained, leaving a large area of necrotic tissue with no healthy corium visible (Fig. 5)

In the mild and moderate cases no distinct smell was detected. There was no underrunning of horn detected until the more advanced stages. In the earlier stages the horn seemed to have been lysed instead of underrun.

In the majority of cases the feet were trimmed and the lesions sprayed once with oxytetracycline spray (‘Engemycine Spray’, MSD Animal Health, Milton Keynes, UK) on both farms. Occasionally the lesions would be covered with a 0.5 cm thick layer of copper sulphate crystals (25 % Cu content, various suppliers) and the foot bandaged for 2 or 3 days. Injectable antibiotics were not used during our visits but had been used previously and were reported to have low success rates (‘Oxytetrin LA’, 200 mg/ml, 1 ml/kg intramuscular, MSD Animal Health, Milton Keynes, UK). Herd treatments using zinc sulphate foot baths once every 2 weeks (‘Golden Hoof’ at 10 % concentration, Shep Fair Animal Health, Abergavenny, UK) were reported to help keep the problem under control although did not prevent new cases. Neither farm kept records of the treatments administered to individual animals so it was not possible to see the progression of cases. Farm staff on both farms, however, felt that the recovery rates for all lesions and treatments were extremely low at less than 20 %.

## Differential diagnosis

As the lesions that were seen on the farm did not resemble footrot, the diagnosis after clinical examination remained unclear. Footrot, treponeme infection, laminitis and primary claw horn lesions with secondary infections were still considered as possible diagnoses. Additional diagnostics were carried out in order to differentiate further.

## Further diagnostics

### Swabs for processing by PCR

During the clinical examination dry swabs were taken from the affected area (interdigital space, exposed corium or laminae) of 20 lame, lactating animals on Farm 1, 16 lame, lactating animals on Farm 2 and two non-lame youngstock on Farm 2. These were taken after the farm staff had finished trimming the feet and before treatments were administered. The swabs were placed in Eppendorf tubes containing 0.5 ml of sterile TRIS EDTA Buffer (pH 8.0) during transport and processed by polymerase chain reaction (PCR) at the laboratory facilities of the University of Bristol. PCR primers used are shown in Table 4.

**Table 4:** PCR primers used

Primer specificity	Primer (sequence)	Predicted size (bp)	Reference
*D. Nodosus*	C TCGGTACCGAGTATTTCTACCCAACACCTAc 50 CGGGGTTATGTAGCTTGC	783	[35]
Spirochaete specific 16S	RNAF AGAGTTTGATCMTGGCTCAGRNAR ACGGCTACCTTGTTACGACTTCAC	1500	[36]
Treponeme specific 16S	TPF AARCATGCAAGTCGARCGGCAAGTPR1 TCCATTGCGGAATATTCTTA	335	[37]

All swabs were found to be negative on PCR for *D. nodosus*. All of the swabs tested positive however on PCR for treponemes. On Farm 2, two samples were taken from non-lame youngstock which also tested negative for *D. nodosus* and positive for treponemes.

### Nutritional breakdown

Both farms used the same concentrate feed which formed approximately 70 % of the goats’ diet (BOCM Pauls Ltd., Ipswich, UK). Nutrient content, as supplied by the manufacturer is shown in Table 5.

**Table 5:** Diet as formulated to contain

DM38 (%)	87.8
Protein (% as fed)	18.2
Starch (% as fed)	10.7
Sugar (% as fed)	7.9
Oil (% as fed)	4.9
NCGD^a^ (% DM)	75.7
NDF^b^ (% DM)	39.3

^a^Neutral cellulase gamminase digestibility

^b^Non detergent fibre

This concentrate was being fed truly ad libitum with either grass hay, haylage or silage offered ad libitum. Although the goats were housed on barley straw this was not intended to constitute a significant amount of the diet. On both farms the quality and type of the forage offered varied greatly between visits. The feeding regime did not differ depending on stage of lactation, production level or dry period. Prior to weaning, the kids would be offered the same concentrates ad libitum. Post weaning, they were fed the same diet as the adult goats.

### Rumen pH

As the nutrition was thought to play a role in the development of the lesions seen, transcutaneous rumen samples were taken from a selection of mid lactation goats, regardless of whether or not they were lame. The samples were taken from the ventral rumen, using a technique described for cattle [24]. The location used was on a line with the dorsal edge of the patella and approximately 10 cm caudal to the last rib. A minimum of 1 ml was obtained from each animal and analysed for pH on farm using the Checker Portable pH Meter (Hanna Instruments, Woonsocket, USA). A total of 18 rumen samples were taken on Farm 1 and 22 from Farm 2 (Table 6). Of the samples taken 17.5 % were below value 5.5, which is considered to be acidotic [25, 26].

**Table 6:** Rumen pH results

PH	Farm 1 [n = 18 (%)]	Farm 2 [n = 22 (%)]
<5.5	3 (16.7)	4 (18.2)
>5.5 to <5.8	6 (33.3)	3 (13.6)
>5.8	9 (50)	15 (68.2)

## Diagnosis and discussion

### Microbial factors

On clinical examination, it became apparent that the cause of lameness in these goats was located in the foot. In sheep flocks where the majority of foot lameness is caused by footrot, *D. nodosus* is found on the feet of both healthy and lame sheep [21]. In the cases described above, there was no evidence of *D.nodosus*.

All samples tested however, were positive for treponemes, suggesting a potential role for these bacteria in the aetiology of lameness on the two study farms. CODD was first described in sheep in 1997 [27] with spirochaetes cultured from lesions of affected animals [28]. These spirochaetes were subsequently speciated as treponemes and found to be the same as the treponemes involved in the aetiology of BDD [29]. The presence of the treponemes on swabs from the youngstock indicate that the youngstock carry the same bacteria. Neither farm practiced a high level of biosecurity between youngstock and adults. Although the groups were not in physical contact, farm staff would readily walk between groups and the same equipment was used. It is interesting to note, however, that no lameness was observed in the youngstock.

In cattle lameness, recent findings suggest that treponemes are involved in ‘NhWL’ [22]. These lesions are typically progressive, painful lesions that involve infection of the corium. Treatment is often unsuccessful [30]. It is hypothesised that in the cases described here the treponemes are in fact a secondary invader of exposed or compromised corium or laminae. This would provide an explanation as to why the lesions are not seen in the youngstock. The youngstock are not exposed to the same risk factors as the lactating does and therefore do not develop the claw horn lesions that seem to precede the infected lesions. Moore et al. [21] also report the presence of treponemes on 38 % of healthy sheep feet, suggesting other factors than only the presence of the bacteria play a role in developing disease.

### Physiological and nutritional factors

As housing and feeding regime were similar between the youngstock and adults, the main difference between the groups was that the adults were lactating. As both calving and metabolic stress can have a negative impact on hoof health in cattle [18, 31], this was considered to be of significance in these goat herds. To date, no work has been done to investigate whether or not kidding has the same effect on the suspensory apparatus of the goats’ foot as it does on cows’ feet. It seems likely, however, that the hormonal changes that might be involved with the weakening of the suspensory apparatus in cattle would have the same effect within a goat [31]. When kept within their usual housing environment the goats normally have soft underfoot conditions. They should therefore not experience the same trauma described in dairy cattle that results from prolonged periods standing on concrete [32]. However, in situations where the quality of the hoof horn is reduced, the foot may be more sensitive to trauma. It is known that the feeding of high levels of non-structural-carbohydrates which are rapidly degraded in the rumen, such as starch, increases the incidence of lameness in dairy herds [18, 33]. Presently, the direct link between acidosis and claw horn lesions is unclear but associations have been made with the production of lesser quality horn [18]. Both herds were fed on the same ad libitum concentrate diet and levels of ruminal acidosis were investigated in the lactating herds. The high concentrate ration fed to both herds was suspected to induce either chronic or sub acute ruminal acidosis (SARA). It must be noted that the diets were formulated to contain high density short fibre from by-products in replacement of forage non detergent fibre (NDF). However, the varying type and quality of forage offered was thought to influence intake and therefore rumen pH might fluctuate considerably. Rumen pH of youngstock was not determined and so greater buffering or diet selection in this group could not be ruled out, potentially limiting acidosis in this group. Although the role of nutrition in the aetiology of cattle claw horn lesions still leaves many questions [18] it is thought that in the current case nutritional factors could not be ruled out. Indeed, when analysing the rumen pH samples, 40 % of samples taken were found to be below 5.8 with 17.5 % below 5.5, a level which in cattle is believed to be indicative of ruminal acidosis [34]. The optimal ruminal pH for goats is reported to be over six, indicating a level of ruminal acidosis in the lactating does [25, 26]. In goats, little is known about the functionality of the rumen under different values of pH in contrast to the extensive literature on cattle. Bava et al. [20] report the ability of goats to adapt to relatively low rumen pH and show little adverse effect of feeding non-forage based diets during lactation. These studies were short and concentrated on a narrow period of time during peak lactation. No evidence is available on the effect of this feeding regime over an extended period of time, which would include the dry period and transition.

### External risk factors

Risk factors for the development of sole haemorrhaging and white line lesions in dairy cattle include incorrect weight distribution, changes to the pedal bone around calving [31], prolonged periods of standing on concrete and the use of grooved concrete in alleys and tracks [32]. As dairy goats are housed differently to dairy cattle (i.e. on bedding rather than concrete) it may not be surprising that under normal circumstances, hoof horn lesions such as sole ulcers are not reported as a cause of lameness in the UK dairy goat herd. Although white line lesions are common [10], in many cases they are not thought to cause lameness. The goats from Farm 1 always walked on flat surfaces and the time stood or walking on concrete was limited. The animals from Farm 2 however, had to walk up and down a relatively narrow and steep ramp to enter and exit the milking parlour and it is possible this causes bruises and white line lesions within this herd and may contribute to the higher incidence of lameness on Farm 2. The goats on Farm 1 only had to travel over level ground but they had to perform a 180° turn with rough concrete underfoot. In dairy cattle rough concrete is demonstrated as a risk factor for white line lesions [32].

## Conclusion

A definite diagnosis cannot be made at this stage. It is, however, hypothesised that the aetiology of these lameness cases may be twofold with both nutritional and infectious involvement. A claw horn lesion develops first, either white line lesion or sole ulcer, due to lower quality horn produced as a result of a poor diet and the specific external risk factors listed for each farm. This claw horn lesion subsequently becomes infected with treponemes, resulting in a painful, progressive lesion that is difficult to cure. Further investigations, including post mortem results, histology and further identification of the treponemes involved, are needed in order to reach a diagnosis. Further work is also required on the impact of extended high concentrate feeding on goat health and specifically lameness.

## Abbreviations

BDD: bovine digital dermatitis 279
CODD: contagious ovine digital dermatitis 280
NhWL: non healing white line lesion 281
NhSU: non healing sole ulcer 282
NCGD: neutral cellulase gamminase digestibility 283
NDF: non detergent fibre 284
PCR: polymerase chain reaction 285
SARA: sub acute ruminal acidosis 286
UK: United Kingdom
